# Supplementary material for: Biodiversity loss reduces global terrestrial carbon storage
Source: Nat Commun. 2024 May 22;15:4354. doi: 10.1038/s41467-024-47872-7 (PMC11111688; doi:10.1038/s41467-024-47872-7)
Supplement: Supplementary file 1 — Supplementary information [file 41467_2024_47872_MOESM1_ESM.pdf]

Biodiversity loss reduces global terrestrial carbon storage

Supplementary Information

Authors List

Sarah R. Weiskopf*[1,2], Forest Isbell[3],Maria Isabel Arce-Plata[4], Moreno Di Marco[5], Mike Harfoot[6], Justin Johnson[7], Susannah B. Lerman[8], Brian W. Miller[9], Toni Lyn Morelli[10,2], Akira S Mori[11], Ensheng Weng[12], Simon Ferrier[13]
*Corresponding author – sweiskopf@usgs.gov

Affiliations

1. U.S. Geological Survey National Climate Adaptation Science Center, Reston, VA;
2. Department of Environmental Conservation, University of Massachusetts, Amherst, MA, USA
3. Department of Ecology, Evolution and Behavior, University of Minnesota, Saint Paul, MN, USA
4. Quantitative & Computational Ecology Laboratory, Département de Sciences Biologiques, Université de Montréal, QC H3T 1J4, Montréal, Canada
5. Department of Biology and Biotechnologies, Sapienza University of Rome, Rome, Italy
6. Vizzuality, 123 Calle de Fuencarral, 28010, Madrid, Spain
7. University of Minnesota, Applied Economics. 1994 Buford Ave. Saint Paul, MN 55105, USA.
8. USDA Forest Service Northern Research Station, Amherst, MA, USA
9. U.S. Geological Survey North Central Climate Adaptation Science Center, Boulder, CO, USA
10. U.S. Geological Survey Northeast Climate Adaptation Science Center, Amherst, MA, USA
11. Research Center for Advanced Science and Technology, University of Tokyo, 4-6-1 Komaba, Meguro, Tokyo, 153-8505, Japan
12. Columbia University/NASA Goddard Institute for Space Studies, 2880 Broadway, New York, NY 10025, USA
13. Land and Water, CSIRO, Canberra, ACT, 2601 Australia

Supplementary Methods: Equations used in the BILBI Model. For additional details, see [1,2]

BILBI is a macroecological model that uses generalized dissimilarity modeling (GDM) to map beta diversity over space and time, meaning that it predicts species turnover based on environmental differences (e.g., climate and habitat variables) and spatial distance between sites [2]. Beta diversity estimates can then be coupled with a modified form of species-area analysis to predict the proportion of species expected to persist within a region of interest (e.g., an ecoregion) under different climate and land-use scenarios [1]. To estimate beta diversity, the model evaluates the probability that a random draw of two species from two sites are the same, which is then converted to a Sorensen dissimilarity index and provides continuous predictions of beta diversity on a 0-1 scale across the area being modeled [1,2].

The following equations are used to predict the proportion of species originally associated with a given grid cell (30 arc-seconds globally, ~1 km2 at the equator) which are expected to persist (i.e. avoid extinction) over the long term, anywhere within their range [1]):

1) Calculate the total area of similar ecological environments relative to a given cell *i* by summing the predicted similarity (under the present baseline climate) to all other cells *j:*

$$A_{i_{\text{baseline}}} = \sum_{j=1}^{j=n} s_{i_{\text{present}} j_{\text{present}}} \quad (1)$$

where $A_{i_{\text{baseline}}}$= the effective area (# of grid cells) of similar ecological environments relative to cell *i* in the baseline period , *n*=the total number of cells for which compositional similarity with cell *i* was modeled, $s_{i_{\text{present}} j_{\text{present}}}$ = the GDM-predicted compositional similarity between cells *i* and *j* (i.e., pairwise beta diversity on a 0-1 scale) under present climatic conditions. Thus, cells that have completely similar composition would be counted as a full grid cell, while less similar cells would count less towards potential area.

2) Calculate the potential area of similar ecological environments under a future scenario ($A_{i_{\text{scenario}}}$):

$$A_{i_{\text{scenario}}} = \sum_{j=1}^{j=n} s_{i_{\text{present}} j_{\text{future}}} h_{j_{\text{scenario}}} \quad (2)$$

where $s_{i_{\text{present}} j_{\text{future}}}$ = the predicted similarity between cell *i* under the present climate and cell *j* under the future climate associated with this scenario (using standard space-for-time substitution), and $h_{j_{\text{scenario}}}$ is the habitat condition score of cell *j* under the scenario.

3) Use the species-area relationship to translate the amount of habitat remaining across similar ecological environments under the scenario (expressed as a proportion of the total area of similar environments prior to climate and land-use change) into the proportion of species (originally associated with cell *i*) expected to persist over the long term ($p_{i_{\text{scenario}}}$):

$$p_{i_{\text{scenario}}} = \left[\frac{A_{i_{\text{scenario}}}}{A_{i_{\text{baseline}}}}\right]^z \quad (3)$$

where *z* is the exponent of the species-area relationship, which determines how loss of species scales with loss of habitat area. A *z* value of 0.25 is widely used in other studies predicting the proportion of species remaining over time in fragmented habitats. However, intact habitats also experience species relaxation (i.e., long-term loss of species as the community approaches equilibrium species richness [3]), commonly estimated at *z*=0.15. To estimate the additional loss of species due to climate and land-use change, we can subtract these two estimates of *z* to obtain a lower bound of *z*=0.1 [4,5]. Other metapopulation models estimate that extinction debts could be higher [5]. Thus, we use a range of z values between 0.1 and 0.65, similar to Isbell et al. (2015), to capture some of the uncertainty around the magnitude of species extinction debts.

We used two scenarios: SSP1/RCP 2.6, a low land-use change and low climate change scenario ("global sustainability"[1]), and SSP5/RCP8.5, a high climate change and intermediate land-use change scenario ("fossil-fueled development") [7,8]. We estimated the collective proportion of those species originally associated with each ecoregion which are expected to persist over the long term as a weighted geometric mean of the p_i values for all cells in the ecoregion. The contribution of each cell was weighted by how unique it is within the context of the biome where the ecoregion is found (i.e., regionally rare

[1] Note that SSP1/RCP2.6 still entails a significant amount of land use change due to bioenergy production and increased food demand [6].

environments have a higher weight, since these areas are likely to have more unique species and thus may contribute more to regional species loss). See [1] for further explanation of this approach.

The proportion of species persisting at the ecoregion level (p_{region}) was therefore calculated as:

$$p_{region} = exp\left(\frac{\sum_{i=1}^{m} w_i ln\,(p_i)}{\sum_{i=1}^{m} w_i}\right) \qquad (4)$$

where *m* is the number of cells in the ecoregion of interest, and w_i is the weight applied to each cell:

$$w_i = \frac{1}{\sum_{j=1}^{j=n} s_{i_{present} j_{present}}}$$

These ecoregional aggregations were performed using the terra package [9] in R version 4.0.4 [10]. $\frac{A_{i_{\mathrm{scenario}}}}{A_{i_{\mathrm{baseline}}}}$ and $s_{i_{present} j_{present}}$ used in this analysis are available to download from figshare [11].

Supplementary Table 1: Projected carbon loss from various sources under global sustainability (SSP1/RCP3.6) and fossil-fueled development (SSP5/RCP8) scenarios.

Source	Timeframe of C emissions	C loss		Other	Citation
		**SSP1/RCP2.6**	**SSP5/RCP8**		
Long-term vegetation carbon loss associated with biodiversity loss	Over the long term (the coming decades) based on cumulative biodiversity loss projected from climate and land-use conditions in 2050.	7.40-102.68 PgC	10.83-145.32 PgC		This analysis
Long-term vegetation and soil carbon loss associated with biodiversity loss	Unclear, but over the long term based on cumulative biodiversity loss projected from climate and land-use conditions in 2050.	18.87 – 262.09 PgC	26.49 – 356.71 PgC		This analysis
Land-use CO2 emissions estimated from integrated assessment models (IAMs) 2006-2100	Cumulative emissions from 2006-2100	61.1 PgC	53.8 PgC		[6] Extracted using WebPlot digitizer from figure 6.23
Emissions from melting permafrost based on IPCC AR6 model ensembles (low confidence)	2100	20-58 PgC	92±17 PgC	3-41 PgC per 1°C warming	[12–14]
LPJ-GUESS	2100		10% increase in ecosystem carbon storage		[15]

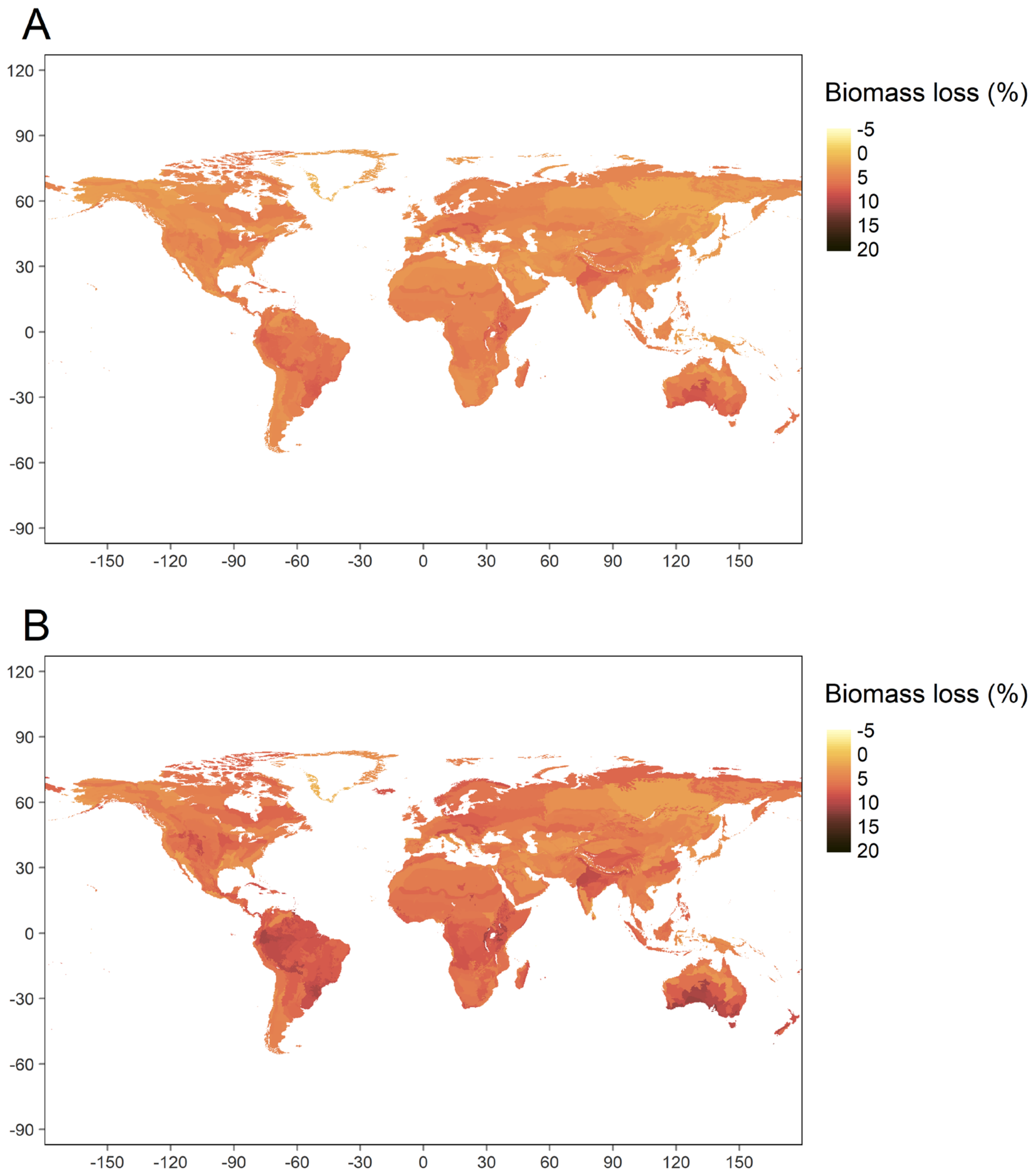

Supplementary Figure 1: Biomass loss by ecoregion - Proportional change in biomass by ecoregion under a global sustainability (SSP1/RCP2.6, panel A) and fossil-fueled development (SSP5/RCP8.5, panel B) scenario using the mean biodiversity-biomass slope b = 0.26 and a species-area relationship of z=0.25. Darker areas indicate greater loss of plant biomass. This biomass loss is from within remaining vegetation as a result of biodiversity loss, over and above any biomass loss resulting from the direct impact of land-use change under a given scenario.

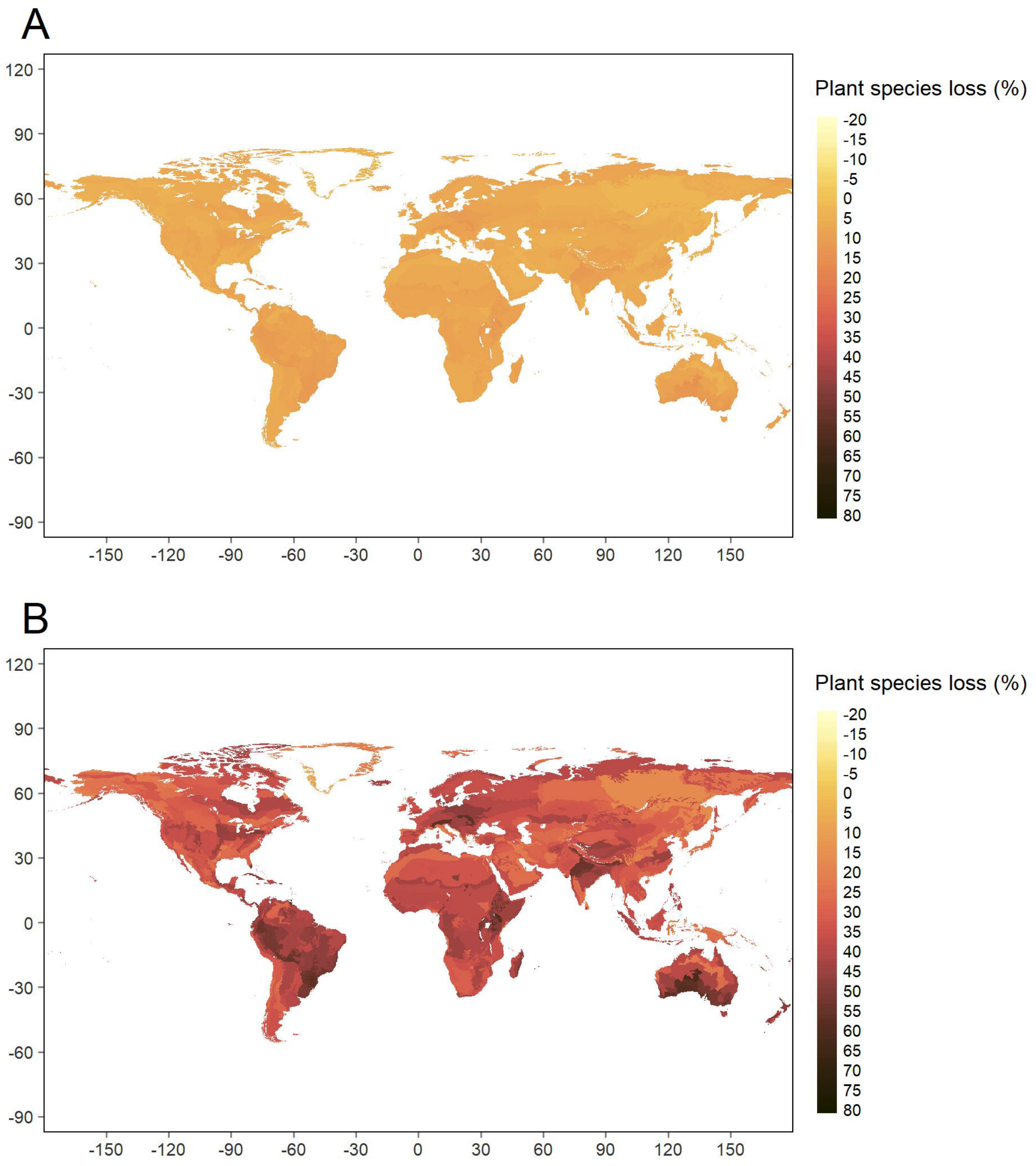

Supplementary Figure 2: Low and high species loss by ecoregion under a global sustainability scenario - The full range of plant species loss by ecoregion projected by the BILBI model under a global sustainability (SSP1/RCP2.6) scenario. Darker areas indicate greater plant species loss. panel A shows plant species loss using the lowest species-area relationship (*z* value) of z=0.10, while panel B shows the highest, z = 0.65.

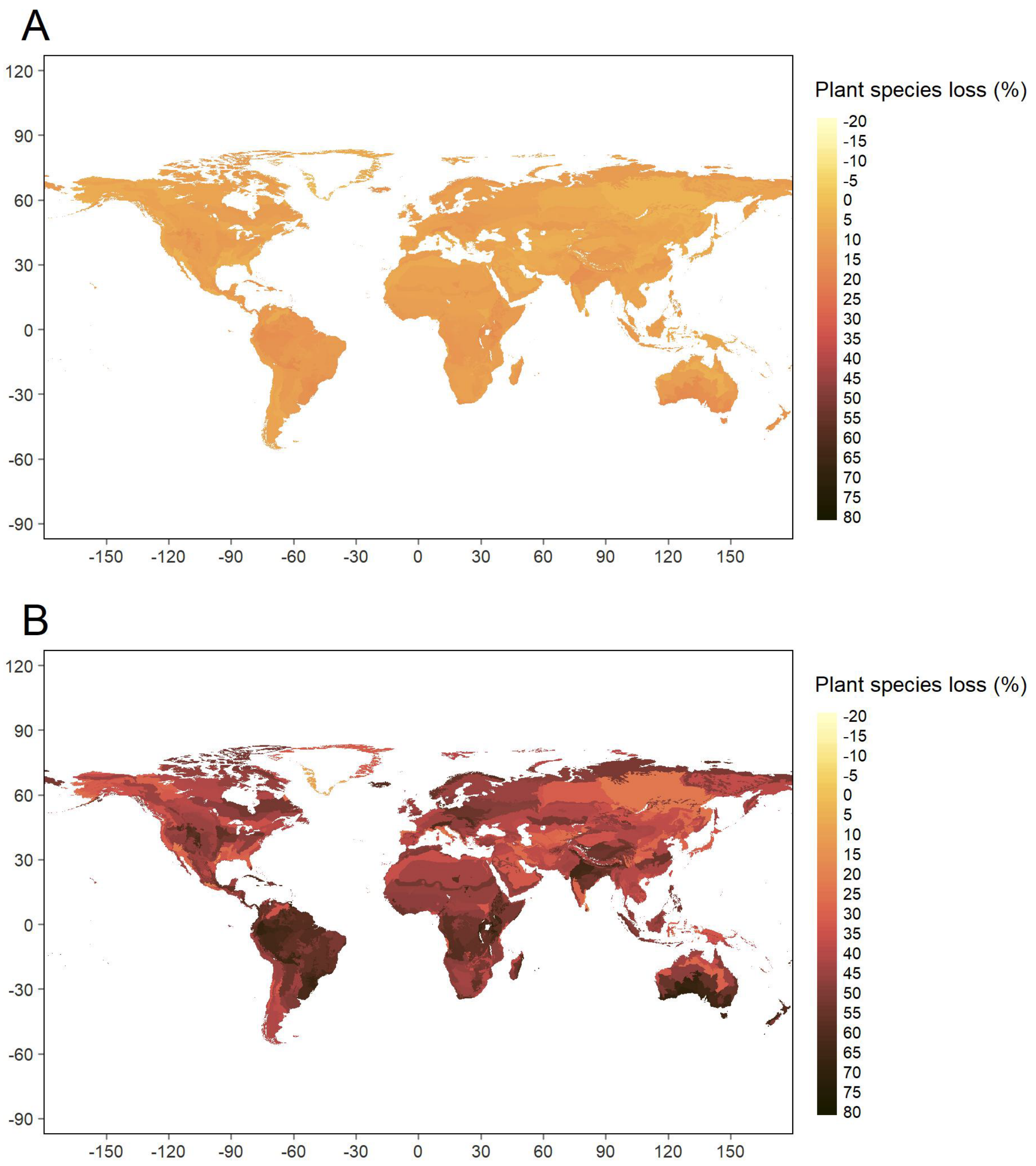

Supplementary Figure 3: Low and high species loss by ecoregion under a fossil-fueled development scenario - The full range of plant species loss by ecoregion projected by the BILBI model under a fossil-fueled development (SSP5/RCP8.5) scenario. Darker areas indicate greater plant species loss. Panel A shows plant species loss using the lowest species-area relationship (*z* value) of *z*=0.10, while panel B shows the highest, *z* = 0.65.

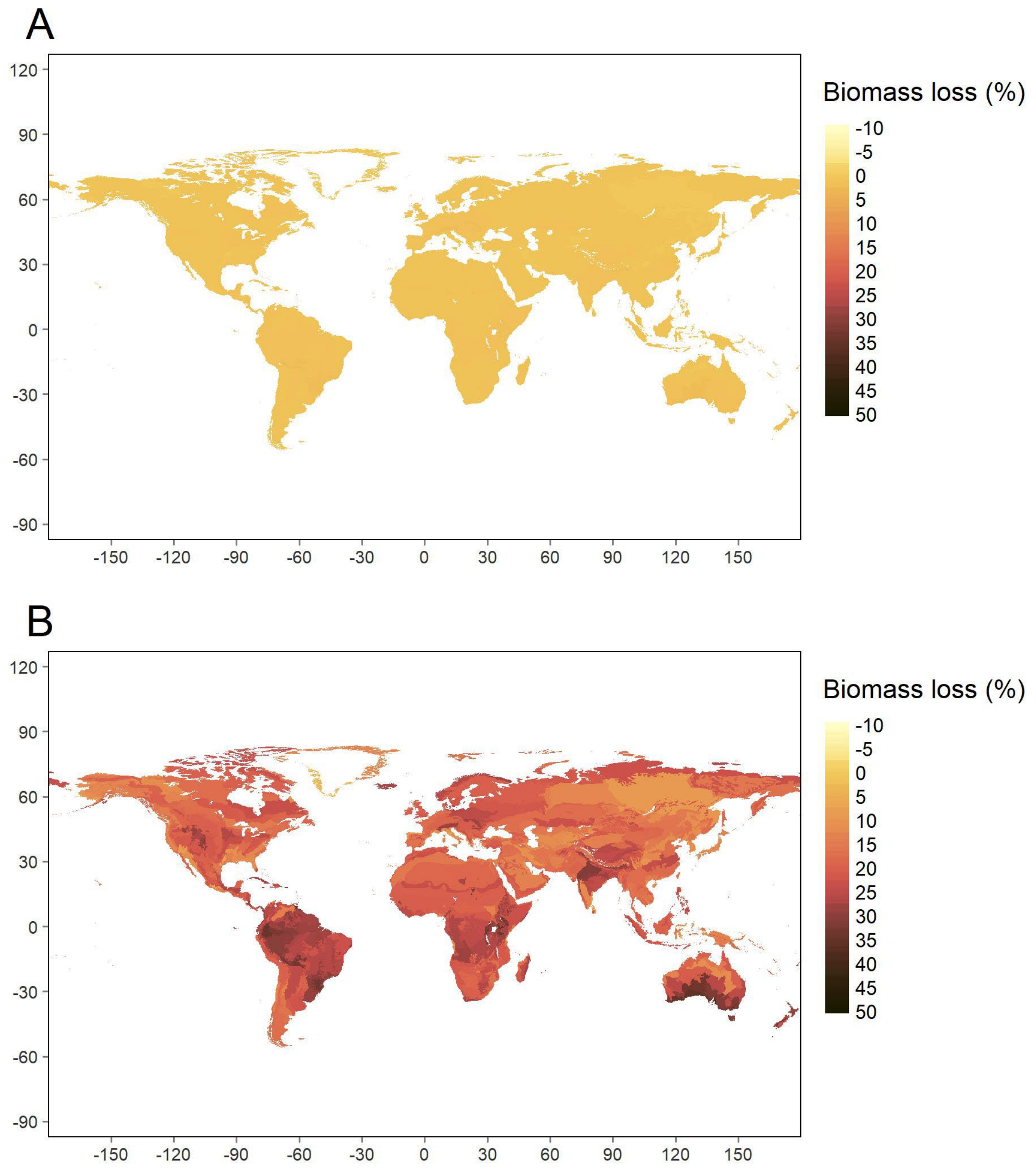

Supplementary Figure 4: Low and high biomass loss estimates by ecoregion- The full range of uncertainty for proportional change in biomass by ecoregion. Darker areas indicate greater biomass loss. Panel A shows proportional biomass loss under a global sustainability scenario using the lowest species-area relationship (*z* value) of *z*=0.1 and the lowest biodiversity-biomass relationship (*b*) of *b*=0.16, while panel B shows the highest of both values under the fossil-fueled development scenario (*z*=0.65, *b*=0.37).

A

B

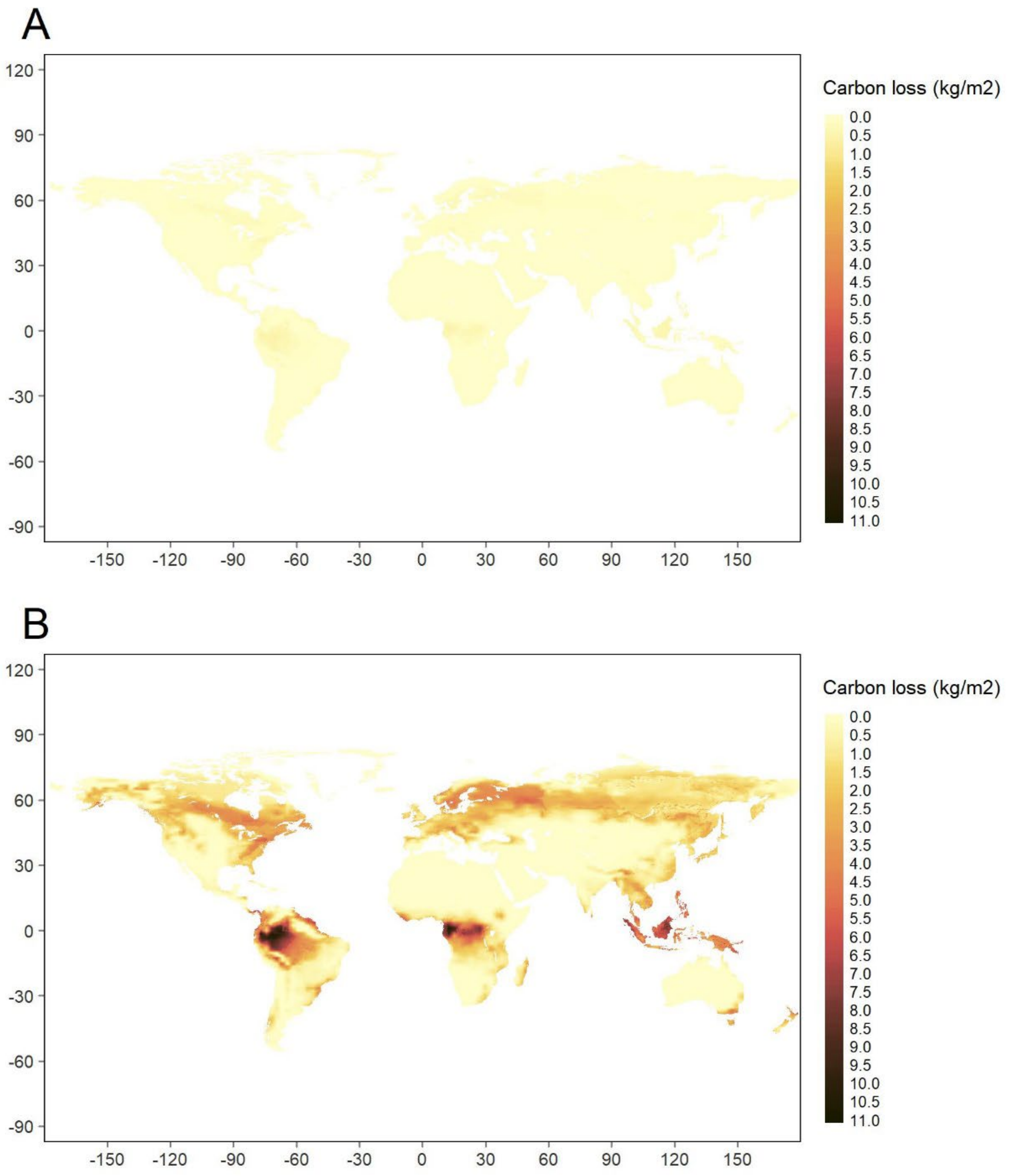

Supplementary Figure 5: Low and high vegetation carbon loss estimates by ecoregion - The full range of uncertainty for vegetation carbon loss from (kg/m^2) driven by long-term loss of plant biodiversity by ecoregion. Darker areas indicate greater percent carbon loss. Panel A shows carbon loss under a global sustainability scenario using the lowest species-area relationship (*z* value) of *z*=0.1 and the lowest biodiversity-biomass relationship (*b*) of *b*=0.16, while Panel B shows the highest of both values under the fossil-fueled development scenario (*z*=0.65, *b*=0.37).

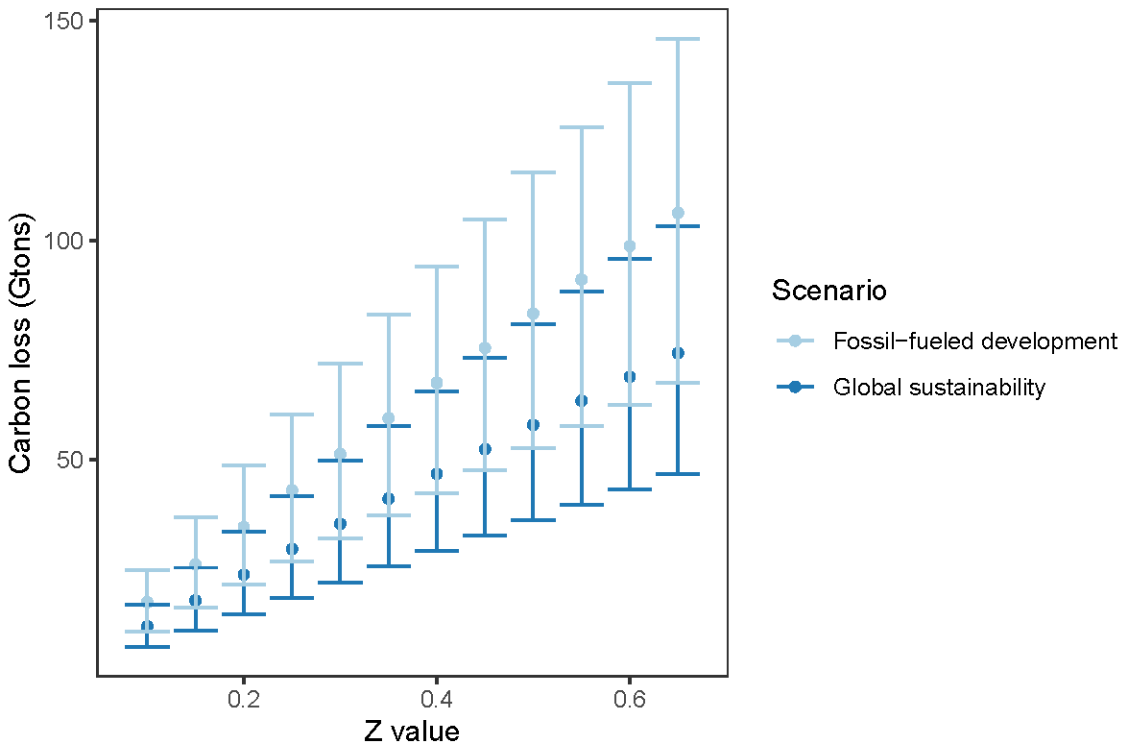

Supplementary Figure 6: **Uncertainty in vegetation carbon loss estimates** – The full range of vegetation carbon loss estimated with different species-area relationships (*z*-values) for different climate and land-use change scenarios. Error bars represent uncertainty across the biodiversity-biomass stock relationship (*b*). Source data are provided as a Source Data file.

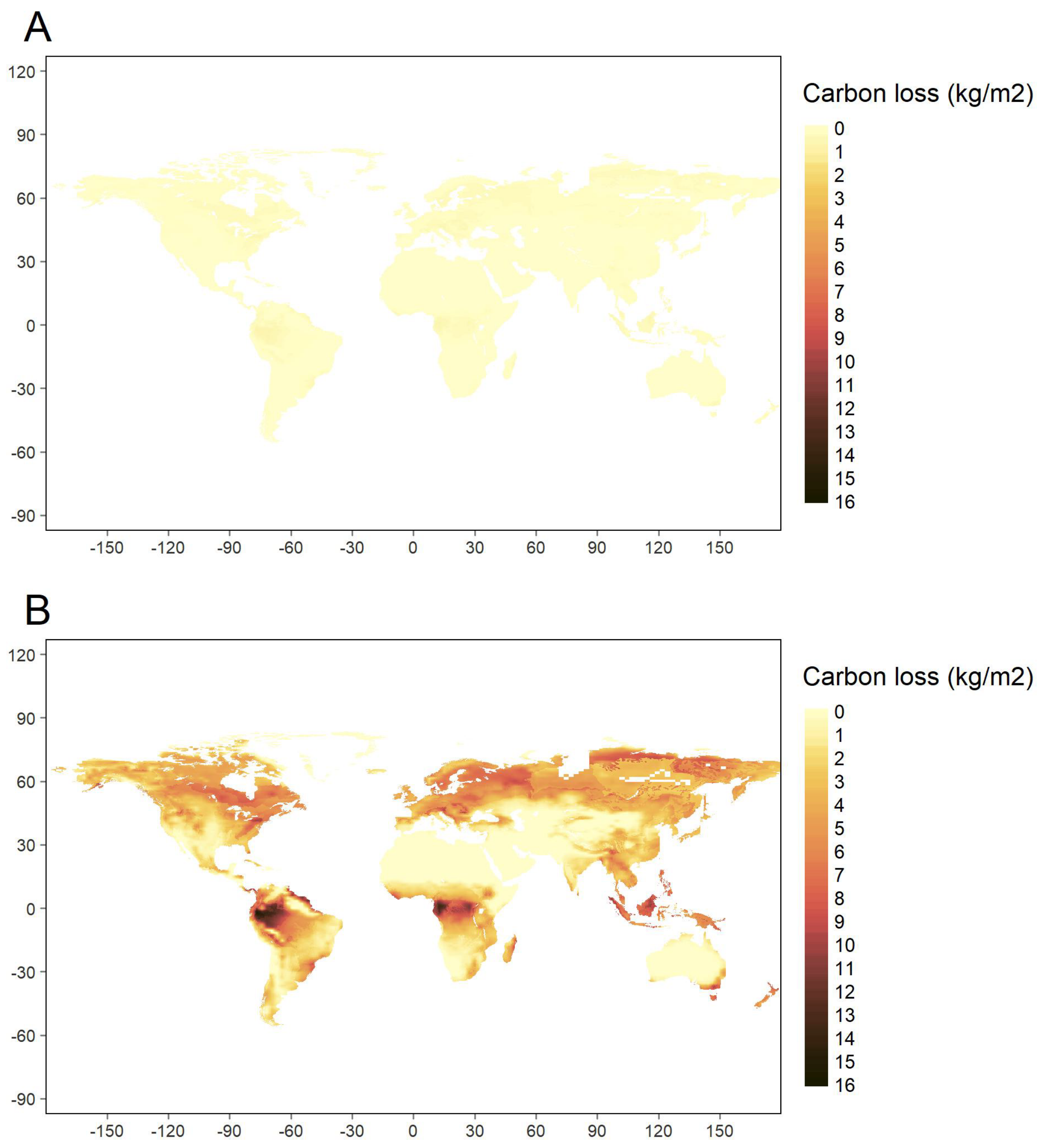

Supplementary Figure 7: **Low and high vegetation and soil carbon loss estimates by ecoregion -** The full range of uncertainty for carbon loss (kg/m^2) driven by long-term loss of plant biodiversity by ecoregion when both vegetation carbon and soil carbon are considered. Darker areas indicate greater percent carbon loss. Panel A shows carbon loss under a global sustainability scenario using the lowest species-area relationship (*z* value) of *z*=0.1 and the lowest biodiversity-biomass relationship (*b*) of *b*=0.16, while panel B shows the highest of both values under the fossil-fueled development scenario (*z*=0.65, *b*=0.37).

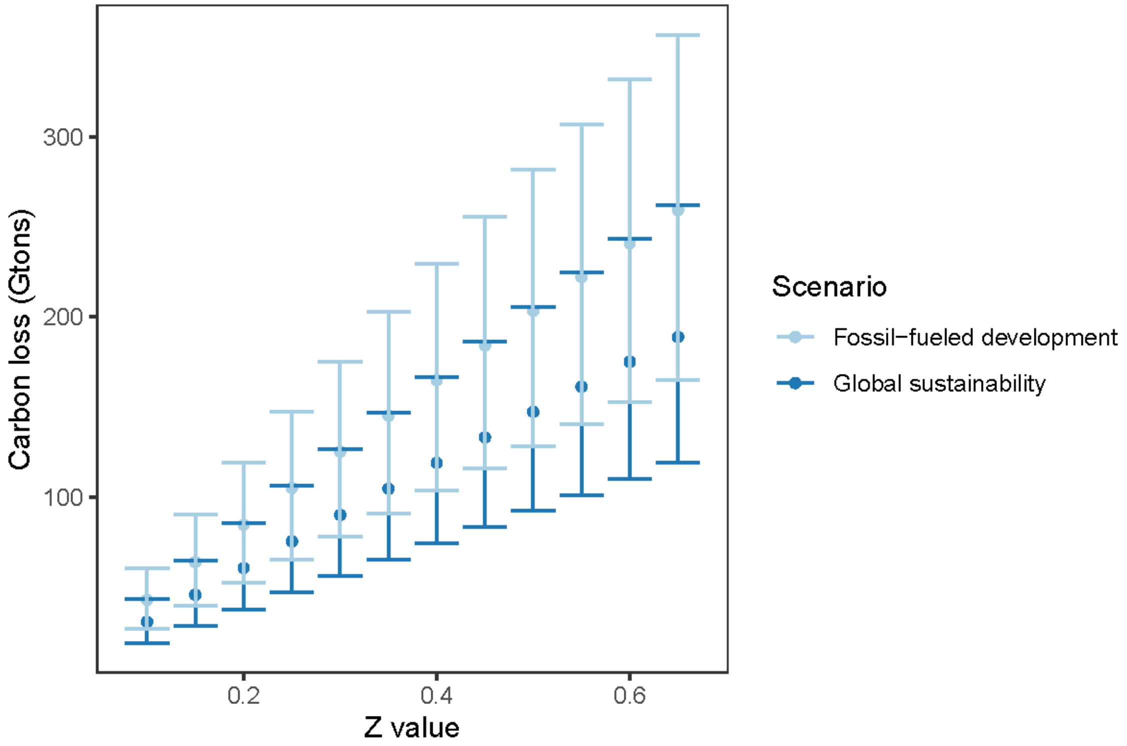

Supplementary Figure 8: **Uncertainty in vegetation and soil carbon loss estimates** – The full range of vegetation and soil carbon loss estimated with different species-area relationships (*z*-values) for different climate and land-use change scenarios. Error bars represent uncertainty across the biodiversity-biomass stock relationship (*b*). Source data are provided as a Source Data file.

Supplementary References

1. Di Marco, M. *et al.* Projecting impacts of global climate and land-use scenarios on plant biodiversity using compositional-turnover modelling. *Global Change Biology* **25**, 2763–2778 (2019).
2. Hoskins, A. J. *et al.* BILBI: Supporting global biodiversity assessment through high-resolution macroecological modelling. *Environmental Modelling and Software* **132**, 104806 (2020).
3. Diamond, J. M. Biogeographic Kinetics: Estimation of Relaxation Times for Avifaunas of Southwest Pacific Islands. *Proceedings of the National Academy of Sciences* **69**, 3199–3203 (1972).
4. Gonzalez, A. Metacommunities: Spatial Community Ecology. *Encyclopedia of Life Sciences* (2009) doi:10.1002/9780470015902.a0021230.

5. Isbell, F., Tilman, D., Polasky, S. & Loreau, M. The biodiversity-dependent ecosystem service debt. *Ecology Letters* **18**, 119–134 (2015).

6. Ciais, P. *et al.* Carbon and Other Biogeochemical Cycles. in *Climate Change 2013: The Physical Science Basis. Contribution of Working Group I to the Fifth Assessment Report of the iItergovernmental Panel on Climate Change* (eds. Stocker, T. F. et al.) 465–570 (Cambridge University Press, 2013).

7. Kriegler, E. *et al.* Fossil-fueled development (SSP5): An energy and resource intensive scenario for the 21st century. *Global Environmental Change* **42**, 297–315 (2017).

8. van Vuuren, D. P. *et al.* Energy, land-use and greenhouse gas emissions trajectories under a green growth paradigm. *Global Environmental Change* **42**, 237–250 (2017).

9. Hijmans, R. J. terra: Spatial Data Analysis. R package version 1.7-39. (2023).

10. R Core Team. R: A language and environment for statistical computing. R Foundation for Statistical Computing (2021).

11. Di Marco, M., Hoskins, A. J., Harwood, T. D., Ware, C. & Ferrier, S. BILBI model data for SSP1/RCP2.6 and SSP5/RCP8.5. figshare https://doi.org/10.6084/m9.figshare.25188650 (2024).

12. Schneider Von Deimling, T. *et al.* Observation-based modelling of permafrost carbon fluxes with accounting for deep carbon deposits and thermokarst activity. *Biogeosciences* **12**, 3469–3488 (2015).

13. Meredith, M. *et al.* Polar regions. in *IPCC Special Report on the Ocean and Cryosphere in a Changing Climate* (eds. Pörtner, H.-O. et al.) (Cambridge University Press, Cambridge, UK and New York, NY, 2019). doi:10.1017/cbo9780511975301.013.

14. Canadell, J. G. *et al.* Global Carbon and other Biogeochemical Cycles and Feedbacks. in *Climate Change 2021: The Physical Science Basis. Contribution of Working Group I to the Sixth Assessment Report of the Intergovernmental Panel on Climate Change* (eds. Masson-Delmotte, V. et al.) 673–816

(Cambridge University Press, Cambridge, UK and New York, NY, 2021). doi:10.1017/9781009157896.007.674.

15. Smith, B. *et al.* Implications of incorporating N cycling and N limitations on primary production in an individual-based dynamic vegetation model. *Biogeosciences* **11**, 2027–2054 (2014).
