# Supplementary material for: Biodiversity loss reduces global terrestrial carbon storage
Source: Nat Commun. 2024 May 22;15:4354. doi: 10.1038/s41467-024-47872-7 (PMC11111688; doi:10.1038/s41467-024-47872-7)
Supplement: Supplementary file 3 — Description of Additional Supplementary Files [file 41467_2024_47872_MOESM3_ESM.pdf]

Description of Additional Supplementary Files

File Name: Supplementary Software 1
Description: Process rasters. R script to turn input raster files into correct format for analysis.

File Name: Supplementary Software 2
Description: Process netcdf. R script to turn netfcdf files into rasters for analysis

File Name: Supplementary Software 3
Description: Generate maps. R script to estimate C loss with confidence intervals for a range of z values

File Name: Supplementary Software 4
Description: Plot maps. R scrip to plot biodiversity, biomass, and carbon loss

File Name: Supplementary Software 5
Description: Carbon accumulation figure. R script to create figure 4.

File Name: Supplementary Software 6
Description: Soil carbon extraction. R script to extract soil carbon for analysis
